# External Validation, Reproducibility, and Interoperability in Digitized Traditional Diagnostic Research: A Cross-Sectional Content Analysis

**DOI:** 10.7759/cureus.115004

**Published:** 2026-08-22

**Authors:** Ankit Srivastava, Vandita Srivastava

**Affiliations:** 1 Homeopathy, Dr. Ankit Srivastava Homeopathic Clinic, Gorakhpur, IND; 2 Department of Sanskrit, Banaras Hindu University, Varanasi, IND

**Keywords:** clinical phenotyping, digital health, electronic health records, external validation, health data, interoperability, machine learning (ml), research reproducibility, traditional medicine

## Abstract

Background: Traditional diagnostic research increasingly uses digital imaging, physiological signals, omics, and machine learning. Technical performance alone, however, does not establish that a method is independently validated, reproducible, or ready for clinical data integration. This study quantified the translational safeguards reported in published human studies of digitized traditional diagnostic phenotypes and signals.

Methods: We conducted a secondary cross-sectional content analysis of a predefined literature library assembled through a documented PubMed and Lens/Scopus search and screening process for a separate scoping-review project and indexed from 2000 through July 4, 2026. This was not a de novo systematic review. From the broader 195-record library, 100 formed the complete source-verifiable analysis frame because all prespecified validation, reproducibility, and interoperability outcomes could be determined from accessible source material at the audit cutoff. Records were not selected according to whether any safeguard was present or absent. Two reviewers evaluated eligibility and prespecified safeguards; 12 content exclusions left 88 eligible studies. We calculated proportions with Wilson 95% confidence intervals (CI) and an explicitly nested clinical-translation cascade.

Results: Internal validation was reported in 51 of 88 studies (58.0%; 95% CI 47.5%-67.7%), whereas eight used an independent external cohort (9.1%; 95% CI 4.7%-16.9%). Prospective clinical evaluation was reported in 10 studies (11.4%), longitudinal outcome validation in two (2.3%), calibration in eight (9.1%), and clinical utility assessment in two (2.3%). Public datasets, analytical code, and accessible trained models were available in 13 (14.8%), three (3.4%), and two (2.3%) studies, respectively. No included study explicitly reported clinical terminology or ontology mapping, electronic health record compatibility, or Fast Healthcare Interoperability Resources mapping. The cumulative cascade was 88 eligible studies, 52 with any internal or external validation, eight with independent external validation, two also prospectively or longitudinally evaluated, one also providing a reusable resource, and none reaching interoperability.

Conclusion: Among the 88 eligible source-verifiable studies included in this complete-case analysis, technical validation was substantially more frequently reported than independent external validation, longitudinal clinical evaluation, reusable code or models, or documented interoperability. These findings characterize the analyzed source-verifiable literature and should not be interpreted as prevalence estimates for the broader 195-record parent library or the field as a whole. Future work should prioritize independent cohorts, clinically meaningful follow-up, reusable research resources, and standardized clinical data representation.

## Introduction

Traditional, complementary, and integrative medicine is increasingly incorporated into research, policy, and digital health agendas. The World Health Organization (WHO)'s Global Traditional Medicine Strategy 2025-2034 emphasizes stronger evidence and appropriate health-system integration, while a joint WHO-International Telecommunication Union technical brief describes rapidly expanding applications of artificial intelligence in traditional medicine [1,2]. These developments create an important distinction: converting a traditional observation into a measurable variable is a technical achievement, but it is not equivalent to demonstrating generalizable clinical value.

Digitization is most visible in automated tongue imaging, physiological-signal analysis, syndrome classification, and molecular mapping. Previous reviews have described advances in image acquisition, color correction, segmentation, feature extraction, classification, mobile platforms, and technical standardization [3-5]. More recent reviews have broadened the discussion to machine learning, knowledge graphs, multimodal data, personalization, and governance [6]. The recurring concern is that impressive development performance may be generated within a single dataset or institution, whereas independent evaluation, clinically meaningful follow-up, reproducible research resources, and implementation-ready data structures remain insufficiently examined.

This gap matters because contemporary reporting and appraisal standards distinguish model development from evaluation and emphasize representative data, transparent reporting, calibration, external validation, open-science practices, and clinical applicability [7-9]. Reusable data and code also support the FAIR principles of findability, accessibility, interoperability, and reusability that underpin modern data stewardship [10]. For traditional diagnostic research, the final step is particularly important: a local image label, syndrome name, or custom software output cannot be reliably exchanged across health systems unless it is represented using recognized terminology and interoperability standards.

We therefore conducted a secondary cross-sectional study-level content analysis of a predefined literature library assembled for a separate scoping-review project; this was not a de novo systematic or comprehensive review of the field. The primary objective was to quantify the reporting of validation, reproducibility, and interoperability safeguards among the source-verifiable studies included in the analysis. Prespecified outcomes included independent external validation, prospective or longitudinal clinical evaluation, publicly reusable data, code, or trained models, and explicit terminology, ontology, electronic health record (EHR), or Fast Healthcare Interoperability Resources (FHIR) implementation. Secondary descriptive objectives were to examine patterns across phenotype domains and publication periods.

## Materials and methods

Study design and source frame

This was a secondary cross-sectional content analysis and meta-research study of published human research. The parent library of 195 records had been assembled through a documented PubMed and Lens/Scopus search and screening process for a separate scoping-review project covering records published from January 1, 2000, through July 4, 2026. The present study analyzed this predefined library to answer a distinct research question and was not intended as a de novo systematic review.

The 100-record source frame comprised the complete set of records for which all prespecified validation, reproducibility, and interoperability outcomes could be determined from accessible source material at the audit cutoff. Records were not selected according to whether any safeguard was present or absent. The remaining 95 records in the broader library were not coded as negative outcomes and were outside this complete-case analysis. The study flow was as follows: broader curated library (n=195), source-verifiable analysis frame (n=100), content exclusions (n=12), and final analytic denominator (n=88). The complete study-level audit, exclusion register, source URLs, and reproducible analysis code are publicly available in Zenodo (DOI: 10.5281/zenodo.21794189).

Parent-library search provenance

The predefined 195-record parent library originated from a broader scoping-review search completed through July 4, 2026. The core PubMed search retrieved 2,781 records and the Lens/Scopus-derived search retrieved 29,548 records; cross-source integration and deduplication produced 31,575 unique records. Phenotype-validation triage retained 911 candidate records, from which 195 studies were finally included in the parent library. These upstream counts describe the construction of the parent library; the present secondary analysis begins with the predefined 195-record library and uses the source-verifiable complete-case frame described below. The exact executed PubMed and Lens/Scopus-derived search strings, date limits, execution dates, and record counts are reproduced in Appendix A.

Eligibility criteria

A study was eligible if it reported original human research in which an identifiable traditional diagnostic phenotype or signal was directly measured, classified, biologically mapped, or clinically validated. Eligible constructs included traditional Chinese medicine syndrome or Zheng categories, instrument-derived or clinician-assessed tongue features, traditional pulse signals, Ayurveda Prakriti or constitution, and other structured traditional diagnostic patterns.

We excluded treatment-efficacy or treatment-response studies when the traditional phenotype was used only to select a therapy, define a treated subgroup, or measure response. We also excluded generic oral, saliva, tongue-coating, wearable-signal, or biomarker studies that did not evaluate a recognizable traditional diagnostic construct; reviews; non-human studies; and computational pharmacology without human diagnostic phenotype data.

Outcome definitions

The primary outcomes were independent external validation; prospective clinical evaluation; longitudinal outcome validation; direct public availability of an analysis-relevant dataset, analytical code, or trained model; explicit clinical terminology or ontology mapping; and EHR or FHIR compatibility. Secondary outcomes were internal validation, calibration, and clinical-utility assessment.

Internal validation included explicit cross-validation, bootstrapping, held-out same-source testing, an internal test set, or psychometric or test-retest validation. Independent external validation required evaluation in a clearly separate institution, geography, or non-overlapping temporal cohort that had not been used for model development or tuning. Calibration required a calibration curve, calibration slope or intercept, Brier score [11], Hosmer-Lemeshow goodness-of-fit assessment [12], or an equivalent method; the area under the receiver operating characteristic curve alone was insufficient.

A public dataset, code repository, or trained model was coded as present only when it was directly accessible. “Available upon reasonable request” was coded as absent. Use of electronic medical records did not constitute interoperability unless the study explicitly implemented a recognized terminology, ontology, EHR exchange model, or FHIR resource or profile.

Review and data extraction

Two authors independently reviewed the source records and resolved differences by consensus. Extraction was study-based rather than model-based: each publication contributed one analytic unit even when it reported multiple algorithms. Study identifiers were preserved across the analytic dataset, exclusion register, summary tables, and clinical-translation cascade. Every included and excluded record was linked to a primary-source URL and a concise evidence note.

Coding and extraction rules

Coding used prespecified operational definitions. Internal validation required explicit cross-validation, bootstrapping, held-out same-source testing, test-retest or psychometric validation, or a distinct internal test set. Independent external validation required evaluation in a clearly separate institution, geography, or non-overlapping temporal cohort that had not been used for model development or tuning. Prospective clinical evaluation required prospective recruitment or data collection used to evaluate the phenotype or associated model, whereas longitudinal outcome validation required association with or prediction of a future clinical outcome using follow-up data. Calibration required a calibration curve, calibration slope or intercept, Brier score, Hosmer-Lemeshow assessment, or an equivalent method; discrimination measures alone were insufficient. Clinical utility required an explicit decision-curve, impact, workflow, patient-benefit, or implementation-utility evaluation. A public dataset or analytical code was coded as present only when directly accessible through a repository or accession; “available on request” was coded as absent for public availability. A trained model required directly accessible weights, an executable model, or another deployable implementation. Interoperability required explicit mapping to a recognized terminology or ontology or implementation of an EHR exchange model or HL7 FHIR resource/profile. Full-text and supplementary material were checked for each applicable outcome; reporting silence after adequate checking was coded as absence, while genuinely inaccessible, contradictory, or ambiguous evidence was not converted into a positive finding. The operational definitions are reproduced in Appendix B.

Statistical analysis

We summarized categorical outcomes as counts and percentages using the final eligible denominator. Wilson 95% confidence intervals (CI) [13] were calculated for the primary and secondary proportions. Phenotype-domain and publication-period comparisons were descriptive. No inferential hypothesis testing was performed because the objective was to quantify reported translational safeguards rather than estimate a treatment effect.

The analysis assessed the reporting of translational safeguards rather than the methodological quality or risk of bias of each study. No single established risk-of-bias instrument was applicable across the heterogeneous biological-mapping, scale-development, association, and prediction-model studies included in the analysis.

The clinical-translation cascade used explicitly nested criteria. It began with all eligible studies, followed by studies with any internal or external validation, studies with independent external validation, studies that also reported prospective or longitudinal evaluation, studies that additionally provided a directly reusable dataset, code, or model, and studies that further implemented terminology, ontology, EHR, or FHIR interoperability.

The cascade was used solely as a descriptive framework and was not intended or validated as a score of “translation readiness.” Progression through its sequential stages should not be interpreted as demonstrating clinical readiness because external validation, prospective evaluation, reproducibility, and interoperability represent related but distinct safeguards.

All source-frame checks, outcome counts, Wilson CI, and cascade calculations were reproduced using the Python script deposited with the study-level dataset in Zenodo (DOI: 10.5281/zenodo.21794189). The final reproducibility check was executed using Python version 3.13.5 (Python Software Foundation, Wilmington, Delaware, United States). The revised study figures were generated using Matplotlib version 3.10.8 (Matplotlib Development Team, open-source software).

Ethics and artificial intelligence disclosure

The study used published study-level information only and did not involve identifiable patient-level data, human biospecimens, or direct participant contact; institutional ethics approval and informed consent were therefore not required.

OpenAI ChatGPT was used to provide assistance with literature-source retrieval, data-management code, statistical programming, preliminary extraction organization, language drafting, and formatting. All eligibility decisions, extracted values, source citations, analyses, interpretations, and final manuscript text were independently verified and approved by the authors, who take full responsibility for the work. The study figures were generated directly from the verified numerical dataset using Matplotlib and were not created or modified using a generative image system.

## Results

Study selection and characteristics

**Figure 1:**
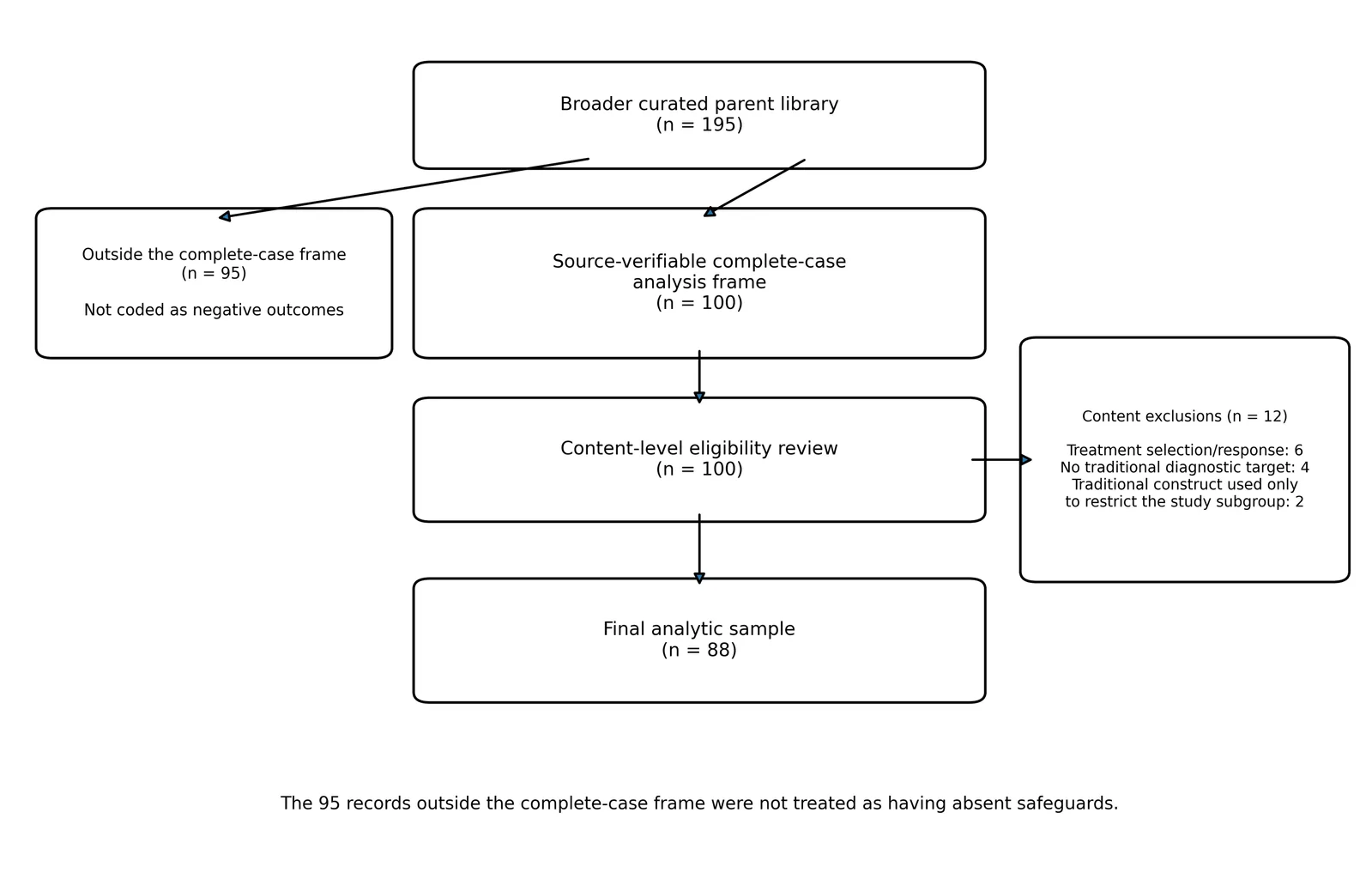
Study selection for the secondary complete-case content analysis The predefined parent library contained 195 records. One hundred records formed the source-verifiable complete-case analysis frame, while 95 records outside this frame were not coded as having absent safeguards. Content-level eligibility review excluded 12 records, leaving 88 studies in the final analytic sample.

All 100 source candidates underwent content-level verification. Twelve records were excluded: six were treatment-focused studies in which the traditional phenotype was used for treatment selection or response assessment, four lacked a traditional diagnostic phenotype or signal as the analytic target, and two used a traditional construct only to restrict the study subgroup. The final analytic denominator was 88 studies (Figure 1).

More than half of the eligible studies were published from 2024 through 2026 (47 of 88, 53.4%). Traditional Chinese medicine syndrome or Zheng studies represented 43 of 88 (48.9%), tongue-diagnosis studies 34 of 88 (38.6%), Ayurveda Prakriti or constitution studies seven of 88 (8.0%), and pulse or mixed-phenotype studies four of 88 (4.5%) (Table 1).

**Table 1:** Characteristics of the 88 eligible studies Percentages use the final analytic denominator of 88 studies. TCM, traditional Chinese medicine

Characteristic	Category	Studies, n	Percentage
Publication period	Up to 2019	16	18.20%
	2020-2023	25	28.40%
	2024-2026	47	53.40%
Phenotype domain	TCM syndrome/Zheng	43	48.90%
	Tongue diagnosis	34	38.60%
	Ayurveda Prakriti/constitution	7	8.00%
	Pulse diagnosis/mixed phenotype	4	4.50%

Validation, reproducibility, and interoperability safeguards

Internal validation was reported in 51 of 88 studies (58.0%; 95% CI, 47.5%-67.7%). In contrast, only eight of 88 (9.1%; 95% CI, 4.7%-16.9%) used an independent external cohort. Prospective clinical evaluation was reported in 10 of 88 studies (11.4%), and only two of 88 (2.3%) used longitudinal outcomes. Calibration was assessed in eight of 88 (9.1%), while clinical utility was assessed in two of 88 (2.3%) (Table 2).

**Table 2:** Prevalence of clinical-translation safeguards Percentages use the final analytic denominator of 88 studies. CI, confidence interval; EHR, electronic health record; FHIR, Fast Healthcare Interoperability Resources

Safeguard	Studies, n/N	Percentage	Wilson 95% CI
Internal validation	51/88	58.00%	47.5%-67.7%
Independent external validation	Aug-88	9.10%	4.7%-16.9%
Prospective clinical evaluation	Oct-88	11.40%	6.3%-19.7%
Longitudinal outcome validation	Feb-88	2.30%	0.6%-7.9%
Calibration assessment	Aug-88	9.10%	4.7%-16.9%
Clinical-utility assessment	Feb-88	2.30%	0.6%-7.9%
Public dataset	13/88	14.80%	8.8%-23.7%
Public analytical code	Mar-88	3.40%	1.2%-9.5%
Accessible trained model	Feb-88	2.30%	0.6%-7.9%
Clinical terminology or ontology mapping	0/88	0.00%	0.0%-4.2%
EHR or FHIR compatibility	0/88	0.00%	0.0%-4.2%

Directly accessible datasets were available in 13 of 88 studies (14.8%). Public analytical code and accessible trained models were substantially less frequent, appearing in three of 88 (3.4%) and two of 88 (2.3%), respectively. No eligible study explicitly reported clinical terminology or ontology mapping, EHR compatibility, or FHIR mapping (zero of 88; 95% CI, 0.0%-4.2%).

Cumulative clinical-translation cascade

**Figure 2:**
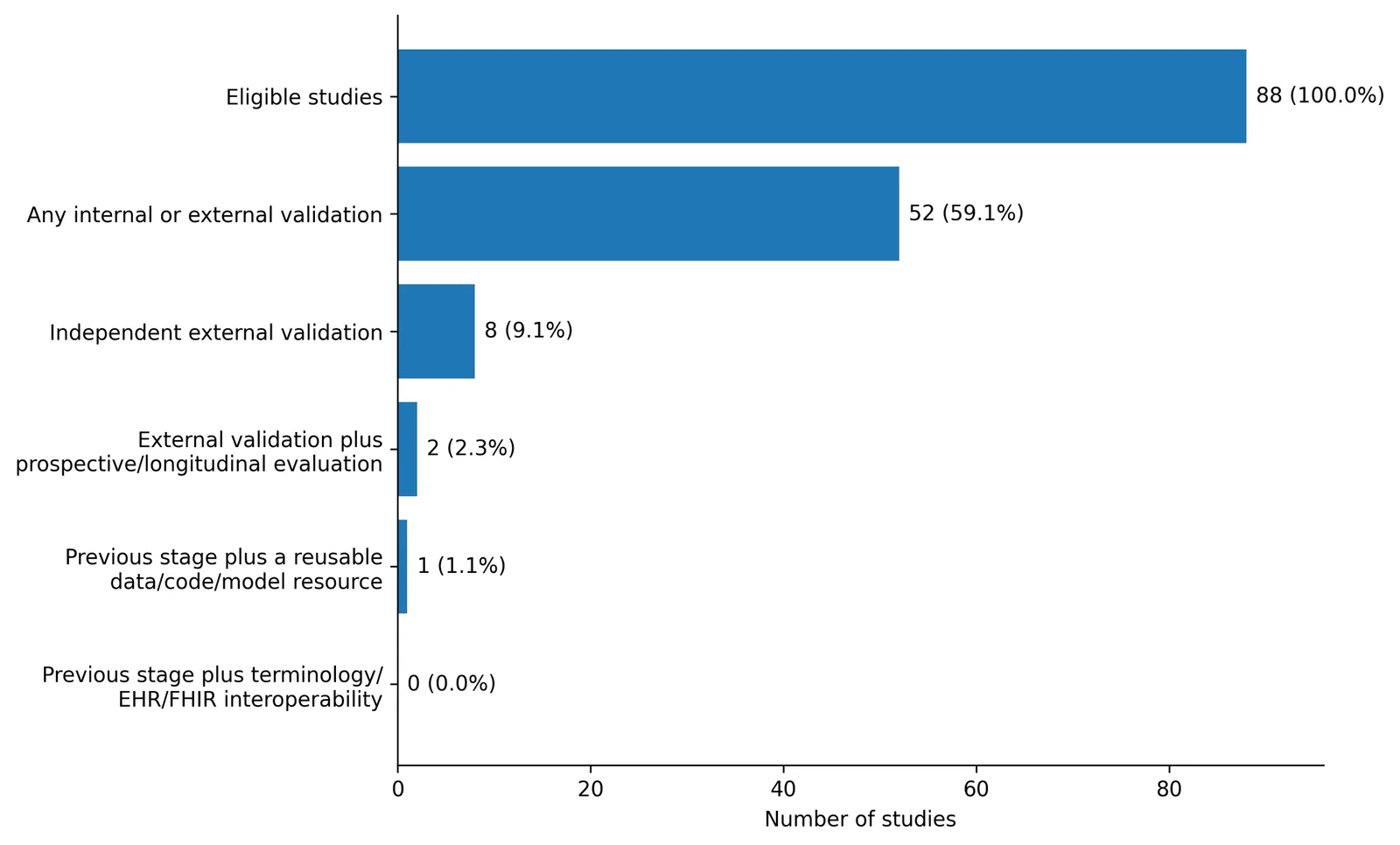
Cumulative clinical-translation cascade Each stage is nested within the preceding stage. Percentages use the final denominator of 88 eligible studies. The figure was generated directly from the verified study-level dataset using Matplotlib version 3.10.8 (Matplotlib Development Team, open-source software). EHR, electronic health record; FHIR, Fast Healthcare Interoperability Resources

The nested cascade narrowed from 88 eligible studies to 52 with any internal or external validation, eight with independent external validation, two that also used prospective or longitudinal clinical evaluation, one that additionally provided a directly reusable dataset, code, or model, and none that further implemented terminology, ontology, EHR, or FHIR interoperability (Figure 2).

Patterns by phenotype domain and publication period

Tongue-diagnosis studies most frequently reported internal validation (25 of 34, 73.5%) and prospective or longitudinal evaluation (nine of 34, 26.5%). Independent external validation remained uncommon across all domains. Among the four pulse or mixed-phenotype studies, all used internal validation, and one used an independent external cohort; the small denominator limits interpretation (Table 3).

**Table 3:** Translational safeguards by phenotype domain Prospective/longitudinal represents the number of unique studies meeting either prospective clinical evaluation or longitudinal outcome validation. The categories were not mutually exclusive: 10 studies reported prospective clinical evaluation, two reported longitudinal outcome validation, and one study met both criteria, yielding 11 unique studies in the combined category. Public resource denotes a directly accessible dataset, analytical code, or trained model. Small-domain percentages should be interpreted cautiously. TCM, traditional Chinese medicine

Phenotype domain	Eligible, n	Internal validation	External validation	Prospective/longitudinal	Any public data/code/model
TCM syndrome/Zheng	43	19/43 (44.2%)	3/43 (7.0%)	1/43 (2.3%)	3/43 (7.0%)
Tongue diagnosis	34	25/34 (73.5%)	4/34 (11.8%)	9/34 (26.5%)	9/34 (26.5%)
Ayurveda Prakriti/constitution	7	3/7 (42.9%)	0/7 (0.0%)	1/7 (14.3%)	1/7 (14.3%)
Pulse diagnosis/mixed phenotype	4	4/4 (100.0%)	1/4 (25.0%)	0/4 (0.0%)	2/4 (50.0%)

Recent publication was associated with greater reporting of technical validation and reusable resources at the descriptive level. Among studies published in 2024-2026, 36 of 47 (76.6%) used internal validation, and 11 of 47 (23.4%) provided at least one public dataset, code repository, or trained model, compared with no public resource among the 16 studies published through 2019. External validation nevertheless remained limited to six of 47 recent studies (12.8%) (Table 4).

**Table 4:** Translational safeguards by publication period Prospective/longitudinal denotes either prospective clinical evaluation or longitudinal outcome validation. Public resource denotes a directly accessible dataset, analytical code, or trained model. Comparisons are descriptive and were not subjected to inferential hypothesis testing.

Publication period	Eligible, n	Internal validation	External validation	Prospective/longitudinal	Any public data/code/model
Up to 2019	16	8/16 (50.0%)	0/16 (0.0%)	0/16 (0.0%)	0/16 (0.0%)
2020-2023	25	7/25 (28.0%)	2/25 (8.0%)	1/25 (4.0%)	4/25 (16.0%)
2024-2026	47	36/47 (76.6%)	6/47 (12.8%)	10/47 (21.3%)	11/47 (23.4%)

## Discussion

Principal findings

Among the 88 eligible source-verifiable studies included in this complete-case analysis, the central finding was a pronounced separation between technical validation and clinical translation. More than half reported some form of internal validation, yet fewer than one in 10 used an independent external cohort. Longitudinal outcome validation, clinical-utility assessment, public code, and accessible trained models were each reported in 3.4% or fewer of the analyzed studies. No included study explicitly reported clinical terminology or ontology mapping, EHR compatibility, or FHIR implementation. These proportions describe the analyzed source-verifiable sample and should not be interpreted as prevalence estimates for the entire underlying literature.

This pattern does not imply that the reported models or biological associations failed. Rather, within the analyzed sample, many studies did not report evidence addressing whether a method would retain performance in a new setting, improve a meaningful clinical decision, remain reproducible outside the originating team, or exchange data with routine health information systems.

Interoperability is the clearest system-level gap

No included study explicitly reported mapping its phenotype or output to a recognized clinical terminology or ontology, and none reported EHR or FHIR compatibility. This should be interpreted strictly as an absence of documented interoperability within the analyzed reports, not as proof that interoperability work, local mappings, or internal coding systems were absent. Reporting practices and the primary purpose of individual studies may also have influenced whether such implementation details were described. Nonetheless, local labels and custom applications remain difficult to compare, query, or exchange without a shared semantic and technical representation.

The infrastructure for this next step already exists. The Traditional Medicine Chapter of the International Classification of Diseases, 11th Revision (ICD-11) provides optional standardized diagnostic categories for international data collection [14]. FHIR defines structured resources for electronic exchange, while SNOMED CT (Systematized Nomenclature of Medicine Clinical Terms) supports consistent semantic representation within EHRs [15,16]. A practical future study does not need to build a new international ontology; it can begin by publishing a reusable phenotype dictionary, documenting mappings to existing classifications where appropriate, and specifying how the variables could be represented in an EHR or FHIR resource.

Technical progress in the analyzed literature has not yet become generalizable evidence

The predominance of internal validation is consistent with a research field that has invested heavily in image processing, feature engineering, omics, and model development [3-6]. Internal cross-validation or a random train-test split can estimate performance within the source population, but it does not test transport to a new institution or population. TRIPOD+AI explicitly distinguishes development data from evaluation data, and PROBAST+AI treats validation design and applicability as central to credible prediction research [7,8]. The STARD-AI guideline similarly emphasizes transparent reporting for artificial-intelligence diagnostic-accuracy studies [9].

The eight externally validated studies demonstrate that stronger designs are feasible. Examples included a prospective tongue-image study in hemodialysis patients with a hospital-specific external test cohort [17], a large prospective multicenter gastric-cancer study with a separate seven-center external cohort [18], and a pulse-wave and vocal-signal model evaluated in an independent external population [19]. These exceptions should become the design target rather than being treated as unusual demonstrations.

Reproducibility remains limited despite recent improvement

Directly reusable research resources were uncommon. Although 14.8% of studies provided a public dataset, only 3.4% provided analytical code and 2.3% provided an accessible trained model. Request-based availability was not counted as public because access depends on author responsiveness and cannot be independently verified. This strict definition is consistent with the FAIR principle that digital research objects should be findable, accessible, interoperable, and reusable [10].

Recent studies provided encouraging examples. One prospective tongue-image project released code and deployed a mobile-compatible model for liver-fibrosis monitoring [20]. However, deployment within a proprietary or local application is not equivalent to providing a transferable clinical-data representation. Future reports should separate public data, analytical code, model weights, acquisition protocols, and deployable clinical interfaces rather than using a single broad data-availability statement.

Implications across traditional diagnostic domains

The analyzed source-verifiable evidence base was not limited to automated tongue imaging. It also included syndrome or Zheng classification, Prakriti assessment, traditional pulse signals, patient-reported syndrome scales, and data-derived pattern rules. Published examples show that structured tools can be developed for Ayurveda Prakriti [21], patient-reported spleen-deficiency syndrome [22], and standardized pattern differentiation [23]. The domain comparison suggests that tongue-diagnosis studies currently have the strongest digital-development infrastructure, but the lack of reported interoperability was shared across every analyzed domain.

A focused translational roadmap

The results support four immediate priorities. First, model-development studies should reserve or recruit a genuinely independent external cohort. Second, diagnostic performance should be connected to prospective or longitudinal outcomes and should include calibration and clinical-utility assessment when appropriate. Third, datasets, code, acquisition protocols, and trained models should be deposited using persistent identifiers. Fourth, phenotype variables and outputs should be accompanied by a reusable dictionary and an explicit terminology, ontology, EHR, or FHIR representation. These steps are conventional clinical-research safeguards, not a new scoring framework specific to traditional medicine.

Strengths and limitations

A principal strength is that the study used direct study-level outcomes rather than an arbitrary composite readiness score. The final denominator, exclusions, counts, CI, source URLs, and cascade are fully traceable in the publicly available study-level dataset and analysis code deposited in Zenodo (DOI: 10.5281/zenodo.21794189). The strict distinction between internal and external validation, request-based and public availability, and local digitalization and interoperability reduces optimistic misclassification.

The study also has important limitations. The analysis was restricted to the 100 records for which all prespecified outcomes could be determined from accessible source material, of which 88 ultimately met content-eligibility criteria. The remaining 95 records in the broader 195-record parent library were outside the complete-case frame and were not coded as having absent safeguards. This source-verification strategy creates a meaningful possibility of availability or selection bias because the 100 source-verifiable records may differ systematically from the 95 records that could not enter the complete-case analysis. Accordingly, the reported proportions describe documented safeguards among the 88 eligible source-verifiable studies analyzed here and should not be interpreted as prevalence estimates for the entire underlying literature. The included studies were also heterogeneous in clinical domain, sample size, measurement platform, study purpose, and methodological maturity, so aggregate estimates may obscure important differences between study types. We assessed whether safeguards were reported, not whether the underlying methods were methodologically adequate or at low risk of bias. No single established risk-of-bias instrument was applicable across the heterogeneous biological-mapping, scale-development, association, and prediction-model studies included in the analysis. Public availability was judged from published reports and accessible links at the time of audit, and repository status may change. Finally, the zero reported interoperability finding reflects explicit reporting and documented implementation in the analyzed articles and does not exclude unreported local mappings or interoperability work outside the scope of the published reports.

## Conclusions

Among the 88 eligible source-verifiable studies included in this complete-case analysis, technical validation was substantially more frequently reported than independent external validation, longitudinal clinical evaluation, reusable code or models, or documented interoperability. These findings characterize the analyzed source-verifiable literature and should not be interpreted as prevalence estimates for the broader 195-record parent library or the field as a whole. Future work should prioritize independent cohorts, clinically meaningful follow-up, reusable research resources, and standardized clinical-data representation.

## Appendices

Appendix A: complete parent-library search strategies

The parent literature library was derived from database searches completed on July 4, 2026, covering publications from January 1, 2000, through July 4, 2026. No language restriction was applied. The PubMed search retrieved 2,781 records, and the Lens/Scopus-derived search retrieved 29,548 records. Cross-source integration and deduplication produced 31,575 unique records before phenotype-validation triage.

PubMed

Exact Executed Search

(("traditional Chinese medicine"[Title/Abstract] OR TCM[Title/Abstract] OR Kampo[Title/Abstract] OR Sasang[Title/Abstract] OR Ayurveda[Title/Abstract] OR Prakriti[Title/Abstract] OR Zheng[Title/Abstract] OR Sho[Title/Abstract] OR "tongue diagnosis"[Title/Abstract] OR "pulse diagnosis"[Title/Abstract] OR "abdominal diagnosis"[Title/Abstract] OR "pattern identification"[Title/Abstract] OR "constitution type"[Title/Abstract]) AND (validat*[Title/Abstract] OR reliab*[Title/Abstract] OR reproducib*[Title/Abstract] OR biomarker*[Title/Abstract] OR genom*[Title/Abstract] OR metabolom*[Title/Abstract] OR proteom*[Title/Abstract] OR transcriptom*[Title/Abstract] OR microbiom*[Title/Abstract] OR "machine learning"[Title/Abstract] OR "artificial intelligence"[Title/Abstract] OR sensor*[Title/Abstract] OR imaging[Title/Abstract] OR physiolog*[Title/Abstract] OR questionnaire*[Title/Abstract])) AND ("2000/01/01"[Date - Publication] : "2026/07/04"[Date - Publication])

Execution date: July 4, 2026. Retrieved records: 2,781.

Lens/Scopus-derived export

Exact Executed Search

TITLE-ABS-KEY(("traditional Chinese medicine" OR TCM OR Kampo OR Sasang OR Ayurveda OR Prakriti OR Zheng OR Sho OR "tongue diagnosis" OR "pulse diagnosis" OR "abdominal diagnosis" OR "pattern identification" OR "constitution type") AND (validat* OR reliab* OR reproducib* OR biomarker* OR genom* OR metabolom* OR proteom* OR transcriptom* OR microbiom* OR "machine learning" OR "artificial intelligence" OR sensor* OR imaging OR physiolog* OR questionnaire*)) AND PUBYEAR > 1999 AND PUBYEAR < 2027

Execution date: July 4, 2026. Retrieved records: 29,548.

Following integration and deduplication, 31,575 unique records underwent phenotype-validation filtering. Context-sensitive triage retained 911 candidate records, and the separate parent scoping-review process ultimately produced the predefined 195-record library used as the starting point for the present secondary analysis.

Appendix B: operational coding and extraction definitions

Each eligible publication constituted one study-level analytic unit. Two authors independently reviewed the source records and resolved disagreements by consensus. Full text, supplementary material, linked repositories, and source URLs were checked when applicable. Reporting silence after adequate checking was coded as absence for reporting outcomes; genuinely inaccessible, contradictory, or ambiguous evidence was not converted into a positive finding.

Internal Validation

Explicit cross-validation, bootstrapping, held-out same-source testing, test-retest or psychometric validation, or a distinct internal test set.

Independent External Validation

Evaluation in a clearly separate institution, geography, or non-overlapping temporal cohort not used for model development or tuning.

Prospective Clinical Evaluation

Prospective recruitment or data collection used to evaluate the traditional diagnostic phenotype or associated model.

Longitudinal Outcome Validation

Association with or prediction of a future clinical outcome using follow-up data.

Calibration Assessment

Calibration curve, calibration slope or intercept, Brier score, Hosmer-Lemeshow assessment, or an equivalent method. Discrimination metrics alone were insufficient.

Clinical-Utility Assessment

Explicit decision-curve, impact, workflow, patient-benefit, or implementation-utility evaluation.

Public Dataset

A directly accessible repository or accession containing analysis-relevant data. “Available on request” was coded as absent for public availability.

Public Analytical Code

A directly accessible code repository containing analysis or model-development code.

Accessible Trained Model

Directly accessible trained weights, executable model, deployable tool, or equivalent reusable implementation.

Clinical Terminology or Ontology Mapping

Explicit mapping to a recognized terminology or ontology, including ICD-11, International Classification of Diseases, 11th Revision (ICD-11), Systematized Nomenclature of Medicine Clinical Terms (SNOMED CT), Logical Observation Identifiers Names and Codes (LOINC), or an equivalent standardized representation.

Electronic Health Record (EHR) or Fast Healthcare Interoperability Resources (FHIR) Compatibility

Explicit implementation of an EHR exchange model or Health Level Seven (HL7) FHIR resource/profile mapping. Use of an electronic medical record without standardized mapping was insufficient.

These definitions are the same operational boundaries used in the study-level audit and final analyses.
